# Reconsidering Case Reports: A Modest Call from a Seasoned Clinician

**DOI:** 10.31662/jmaj.2025-0362

**Published:** 2025-09-12

**Authors:** Shigeki Matsubara

**Affiliations:** 1 Department of Obstetrics and Gynecology, Jichi Medical University, Tochigi, Japan; 2 Department of Obstetrics and Gynecology, Koga Red Cross Hospital, Koga, Japan; 3 Medical Examination Center, Ibaraki Western Medical Center, Chikusei, Japan

**Keywords:** case, case report, concept, journal, original article

## To the Editor

Recently, the *Japan Medical Association* (*JMA*)* Journal* announced it would no longer accept case reports ^(1)^. Considering the increasing number of submissions and limited space, this decision is understandable. This seasoned doctor modestly asks readers and journals to reconsider the value and role of case reports.

To readers, especially the younger generation: please do not assume that case reports are less valuable than original articles. New diseases or innovative ideas often begin with the close observation of a case. In 1908, Takayasu reported an eye lesion of a condition later recognized as Takayasu disease in a case report. I am a retired obstetrics-gynecology professor. In 1997, B-Lynch introduced a new hemostatic procedure for postpartum hemorrhage in a short case series involving five patients: the B-Lynch suture ^(2)^. This marked a Copernican revolution―the uterus should be sutured in an anteroposterior direction with a single thread to achieve hemostasis. Many “modified” procedures have since been published as “original articles,” but the original B-Lynch suture remains the gold standard.

Reflecting on my 46 years of clinical practice, I followed a typical path: first, write a case report ^(3)^, then―often over 10 years later―publish an original article summarizing accumulated cases ^(4)^. That first report launched my lifelong study of each issue ^(3), (4)^. A doctor’s keen eye may detect something new in a single case, and such insights have often been shared through case reports.

To journals: please retain space for such contributions, even if the threshold for acceptance is high. If a formal “case report” section is not feasible, consider flexible formats―e.g., case series written in original article structure or review articles incorporating notable cases. However, some reviewers, especially when strictly adhering to journal regulations, may reject such submissions, insisting that “this should be a case report,” often without carefully evaluating its true significance.

Many journals have discontinued case reports. Dedicated case report journals now exist―some indexed in PubMed―but many charge high publication fees. Yet case reports are often the first papers written by residents, who may be financially constrained. Valuable insights could be lost if they give up writing. Who can deny the possibility that the next Takayasu disease or B-Lynch suture lies hidden in an abandoned draft?

Scientific value does not depend on paper type or length. Watson and Crick’s landmark paper on DNA structure was only one page long ^(5)^. I do not single out the *JMA Journal* ^(1)^, but address medical journals in general.

## Article Information

### Author Contributions

Designed the study, wrote, edited, and approved the final manuscript: Shigeki Matsubara. Met the ICMJE criteria for authorship: Shigeki Matsubara.

### Conflicts of Interest

None

### Ethics Approval

Jichi Medical University does not demand IRB approval for this type of study. Patient anonymity preservation and informed consent for reporting are not applicable.

### Data Availability Statement

Data sharing is not applicable to this article as no new data were created or analyzed in this study.

## References

[1] New policy on case reports and images [Internet]. JMA Journal. 2024 [cited 2025 Jul 21]. Available from: https://www.jmaj.jp/static.php?d=250717&c=news

[2] B-Lynch C, Coker A, Lawal AH, et al. The B-Lynch surgical technique for the control of massive postpartum haemorrhage: an alternative to hysterectomy? Five cases reported. Br J Obstet Gynaecol. 1997;104(3):372-5. 9091019 10.1111/j.1471-0528.1997.tb11471.x

[3] Matsubara S, Kuwata T, Usui R. Forceps holding the cervix for postpartum haemorrhage. J Obstet Gynaecol. 2011;31(6):509. 21823851 10.3109/01443615.2011.584646

[4] Takahashi H, Baba Y, Usui R, et al. Hemostatic effect of combined procedures for placenta previa: cervix-holding, intrauterine balloon, and uterine compression suture. J Matern Fetal Neonatal Med. 2022;35(25):8710-6. 34758709 10.1080/14767058.2021.1999922

[5] Watson JD, Crick FH. Molecular Structure of nucleic acids; a structure for deoxyribose nucleic acid. Nature. 1953;171(4356):737-8. 13054692 10.1038/171737a0

